# Efficacy of terutroban in preventing delayed cerebral ischemia after subarachnoid hemorrhage: a functional isotope imaging study on a rat model

**DOI:** 10.1186/cc13651

**Published:** 2014-03-17

**Authors:** D Lagier, B Guillet, L Velly, N Bruder, M Alessi

**Affiliations:** 1 CHU Timone, Marseille, France; 2 Aix Marseille University, Marseille, France

## Introduction

After a subarachnoid hemorrhage (SAH), delayed cerebral ischemia (DCI) remains the principal cause of morbid mortality. F2-isoprotanes are recognized as biomarkers of DCI. These lipid metabolites, by fixing the thromboxane and prostaglandin (TP) receptor, induce vasoconstriction and platelet aggregation. Our objective was to evaluate the efficacy of terutroban (TER), a TP receptor- specific antagonist, in preventing DCI after SAH.

## Methods

Twenty rats were assigned to one of three groups: a double 250 μ! intracisternal injection (ICI) was realized with saline in the CONTROLS group (*n *= 6) or with autologous arterial blood in the SAH (*n *= 8) and SAH+TER (*n *= 6) groups. Treated animals received an oral administration of 30 mg/kg/day TER during 5 days following blood injection. Rats were evaluated using a functional isotope imaging technique (high-resolution microSPECT). Brain capture of three 99 m technetium radiolabeled tracers was evaluated: HMPAO at day (D) 0, 2 and 5 for cerebral perfusion quantification, DTPA at D3 for blood-brain barrier (BBB) integrity study and annexin V-128 at D4 for apoptotic activity study. Radioactivity was measured in a predefined region of interest: cerebrum, cerebellum and brainstem. Statistical analysis: oneway ANOVA followed by Student's *t *test.

## Results

**Figure 1:**
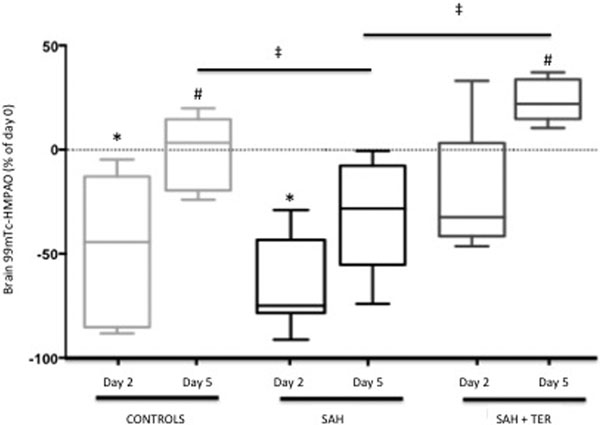
HMPAO uptake at day 2 and day 5. *P <0.01 versus day 0; #P <0.01 versus day 2; tP <0.01.

Brain HMPAO perfusion microSPECT (Figure 1) reveals a transient hypoperfusion after ICI (D2) and a lasting hypoperfusion in the SAH group. TER curtailed the SAH-induced decrease of the HMPAO uptake. TER also significantly counteracted the SAH-induced increase of DTPA in the brainstem (SAH 2.6 ± 0.7 vs. SAH+TER 1.3 ± 0.1 ppm/ mm^3^; *P *< 0.05) and increase of annexin V-128 in the cerebrum (SAH 1.2 ± 0.1 vs. SAH+TER 1 ± 0.06 ppm/mm^3^; *P *< 0.05).

## Conclusion

We made the first microSPECT scan description of a DCI rat model. After induction of SAH, TER improves cerebral perfusion, prevents BBB disruption in the brainstem and decreases apoptotic phenomenon in the cerebrum.

